# Analysis of inter-joint coordination during the sit-to-stand and stand-to-sit tasks in stroke patients with hemiplegia

**DOI:** 10.1186/s13102-023-00716-1

**Published:** 2023-08-16

**Authors:** Jian He, Dongwei Liu, Meijin Hou, Anhua Luo, Shuhao Wang, Ye Ma

**Affiliations:** 1 Research Academy of Grand Health, Faculty of Sports Sciences, Ningbo University, Ningbo, Zhejiang China; 2 School of Information Management and Artificial Intelligence, Zhejiang University of Finance and Economics, Hangzhou, Zhejiang China; 3 National Joint Engineering Research Centre of Rehabilitation Medicine Technology, Fujian University of Traditional Chinese Medicine, Fuzhou, Fujian China; 4 Key Laboratory of Orthopaedics and Traumatology of Traditional Chinese Medicine and Rehabilitation, Fujian University of Traditional Chinese Medicine, Ministry of Education, Fuzhou, Fujian China

**Keywords:** Stroke, Kinematic, Joint coordination, Sit-to-stand, Stand-to-sit

## Abstract

**Background:**

Inter-joint coordination is an important factor affecting postural stability, and its variability increases after fatigue. This study aimed to investigate the coordination pattern of lower limb joints during the sit-to-stand (Si-St) and stand-to-sit (St-Si) tasks in stroke patients and explore the influence of duration on inter-joint coordination.

**Methods:**

Thirteen stroke hemiplegia patients (five with left paretic and eight right paretic) and thirteen age-matched healthy subjects were recruited. The Si-St and St-Si tasks were performed while each subject’s joint kinematics were recorded using a three-dimensional motion capture system. Sagittal joint angles of the bilateral hip, knee and ankle joints as well as the movement duration were extracted. The angle-angle diagrams for the hip-knee, hip-ankle and knee-ankle joint were plotted to assess the inter-joint coordination. The inter-joint coordination was quantified using geometric characteristics of the angle-angle diagrams, including perimeter, area and dimensionless ratio. The coefficient of variation (CV) was performed to compare variability of the coordination parameters.

**Results:**

There were no significant differences in the perimeter, area and dimensionless ratio values of the bilateral hip-knee, hip-ankle and knee-ankle inter-joints during Si-St and St-Si tasks in the stroke group. The perimeter values of bilateral hip-knee and knee-ankle inter-joints in the stroke group were lower (*P*<0.05) than in the healthy group during Si-St and St-Si tasks. Although no significant bilateral differences were found, the inter-joint coordination in stroke patients decreased with the increased movement duration of both Si-St and St-Si tasks. Additionally, the CV of the hip-knee inter-joint area during the Si-St task in the stroke group was less than (*P*<0.05) that in the healthy group.

**Conclusion:**

Stroke patients exhibit different inter-joint coordination patterns than healthy controls during the Si-St and St-Si tasks. The duration affects joint coordination, and inter-joint coordination is limited on the hemiplegic side joint pairs, which may lead to inconsistency in the rhythm of the left and right leg inter-joint movements and increase the risk of falls. These findings provide new insights into motor control rehabilitation strategies and may help planning targeted interventions for stoke patients with hemiplegia.

## Introduction

Rising from a chair and sitting down, called *sit-to-stand* (Si-St) and *stand-to-sit* (St-Si), respectively, are essential activities in daily life and are often performed more than 50 times per day [1, 2]. Si-St and St-Si are demanding biomechanical tasks because they require the coordination of the neuromuscular system to regulate the horizontal, vertical and downward movement of the center of mass and to control postural alignment of the body [3]. After a stroke, the patient’s ability to perform Si-St and St-Si is reduced [4]. Difficulties in performing the Si-St and St-Si activities interfere with the performance of other activities (e.g., walking, standing upright and transferring from bed to chair), leading to a less active lifestyle and deterioration in overall functioning [5–7].

There is evidence from epidemiologic studies that the majority of falls occuring during transfer activities [8]. The off-seat phase of the Si-St movement bears the highest risk of body imbalance [9], indicating that the risk of falling is also the highest during this phase. Similarly, St-Si, as a reverse movement of Si-St, bears the highest risk body imbalance and falls during the contact-seat phase [10]. Most hemiplegic stroke patients perform Si-St and St-Si movements by relying on their unaffected leg due to difficulty in moving their affected leg [11]. Although the left and right legs are used equally in Si-St and St-Si movements, hemiplegic patients use the affected side less during the movements due to muscle weakness [12]. However, this reliance on the non-affected side increases the risk of falls during Si-St and St-Si movements in hemiplegia patients. Falls during Si-St and St-Si tasks may be related to multiple factors, such as inability to counteract unexpected external forces, vestibular impairment, orthostatic hypotension, or deterioration in neuromuscular function [7]. Additionally, falls during Si-St and St-Si movements may also be associated with asymmetric lower limb movement patterns [13, 14].

Previous studies have provided normative data on variables such as force production, joint motion and muscle activity during Si-St and St-Si movements in stroke patients [12, 15–18]. These studies have described the Si-St and St-Si movement patterns and task performance in stroke patients, which can serve as a reference for the development of rehabilitation programs. However, there is little information available on joints coordination during Si-St and St-Si movements. Reisman et al. [19] suggested that coordination differences between Si-St and St-Si may be caused by the length of the support base (e.g., the forefoot is longer than the rear foot), the role of gravity, the required muscle action (e.g., the center versus the eccentricity), and the availability of visual information (e.g., not looking backward while sitting). Shum et al. [20] reported that patients with low back pain had differences in hip-spine coordination during Si-St and St-Si activities. Additionally, a recent study found that Si-St fatigue influenced variability in inter-joint coordination (e.g., hip-knee joint) by in young and older adults [21].

Si-St and St-Si movements require coordination between lower limb joints for balance and stability [22, 23]. The collective actions of the hip, knee, and ankle joints are required during Si-St and St-Si movements [24]. Abnormal joint motion patterns are typically observed in the joint motion of multiple joints rather than in a single joint. Inter-joint coordination can be quantified using a number of techniques that provide varying degrees of complexity and accuracy of results [25]. Kinematic analysis can provide information about the abnormal motion pattern of multiple joints of the lower extremity through inter-joint coordination [26]. The information can be extracted from angle-angle diagrams, which are closed trajectories obtained by plotting the joint angle against other joints simultaneously [27]. Geometric features such as perimeter, area and dimensionless ratio are calculated based on the closed trajectory. These features have been used to evaluate the inter-joint coordination patterns of gait in various diseases such as stroke, knee osteoarthritis and multiple sclerosis [28–30]. However, the mechanism of inter-joint coordination between the left and right legs of Si-St and St-Si movements in stroke patients with hemiplegia, and the effect of motion duration on joint coordination ability, are not fully understood at present.

Understanding the mechanism of inter-joint coordination and differences could improve our knowledge of the relationship between Si-St and St-Si performance and fall risk. This is important for maintaining bilateral coordination movement and preventing falls during Si-St and St-Si transfer in stroke patients with hemiplegia. The aim of this study was to investigate the coordination involving sagittal plane bilateral hip-knee, knee-ankle and hip-ankle inter-joints and the degree of variability in stroke patients with hemiplegia during Si-St and St-Si movements, and to compare the differences with healthy adults. Moreover, we also investigated the effect of the duration of Si-St and St-Si tasks on joint coordination in stroke patients with hemiplegia.

## Methods

### Participants

Stroke patients were included if they met the following criteria were included: (1) stroke was confirmed by CT or MRI medical image, (2) hemiplegia of the lower limbs, (3) between 50 and 80 years old, (4) Functional Ambulation Category Scale (FACS) grades between 2 and 5, (5) no orthopedic surgery or other spasticity treatment within 5 months before experiment, (6) medically stable and able to complete experiment, (7) cognitively normal and able to follow instructions. Inclusion criteria for the healthy subjects were age-matched with the stroke group and had no neuromuscular disorders affecting lower limb functions. Subjects were excluded if they had other serious diseases, underwent surgery on lower extremities, or had communication impairments.

Thirteen patients with stroke hemiplegia (five with left paretic and eighte right paretic) and age-matched thirteen healthy subjects were recruited voluntarily to participate in this study after signing the informed consent form. This study was approved by the Ethics Committee of Shanghai Yangzhi Rehabilitation Hospital (Shanghai Sunshine Rehabilitation Center) with the registration number of 2020-071.

To evaluate the effect of movement duration on hip-knee, hip-ankle, and knee-ankle inter-joint coordination during Si-St and St-Si tasks, the stroke group was further divided into two groups based on the Si-St and St-Si duration characteristics reported in previous studies [4, 24, 31] and combined with the time recorded in our study, as follows:

- Short duration group (duration ≤ 1.6s, *n* = 6) and long duration group (duration > 1.6s, *n* = 7) in the Si-St task.
- Short duration group (duration ≤ 1.9s, *n* = 6) and long duration group (duration > 1.9s, *n* = 7) in the St-Si task.

### Data acquisition

**Fig. 1:**
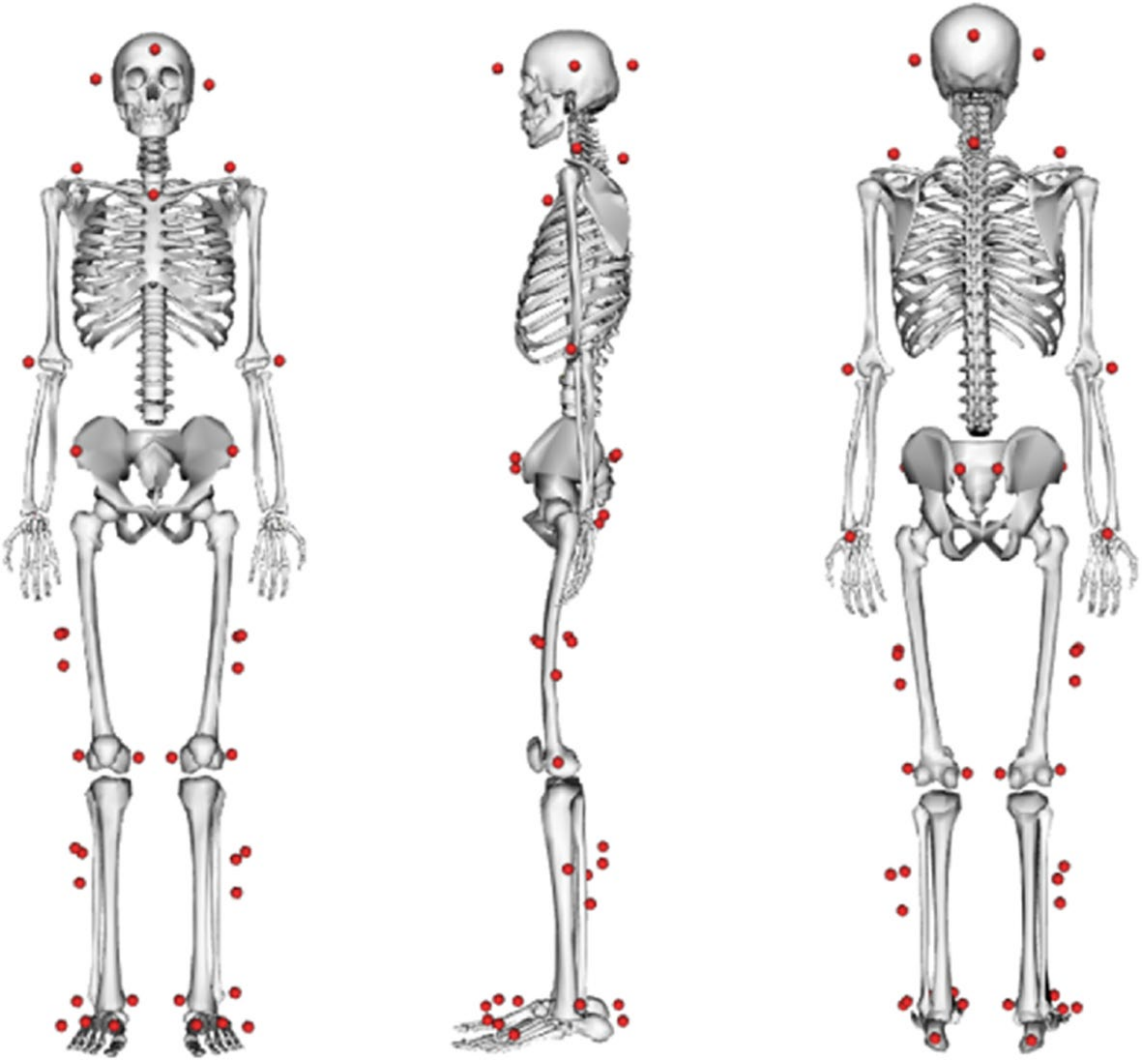
The location of the subject’s experimental markers at the time of data collection

Before experimental collection, we assessed lower limb motor ability of stroke patients using FACS and Brunnstorm scales, as well as spasticity symptoms were assessed using Modified Ashworth scale (MAS). The assessment was conducted by an experienced clinical physiotherapist. We used a three-dimensional motion capture (3DMC) system with eight infrared cameras (VICON Motion Systems Ltd, UK) at a sampling frequency of 100 Hz to collect kinematics data during the Si-St and St-Si tasks. Reflective markers were attached to the head (anterior-posterior and left-right direction), shoulder (bilateral acromion), clavicle, 7th cervical vertebra, hand (bilateral lateral epicondyle of humerus, and wrist), pelvis (anterior and posterior superior iliac spines), thigh (bilateral mid-thigh, and medial and lateral epicondyle of femur), shank (bilateral mid-calf and medial and lateral malleolus), and foot (bilateral heel and the first, third and fifth metatarsal bones) based on the calibrated anatomical system technique (CAST) protocol (see Fig. 1).

The subject naturally stands up and sits down in a backless and armless chair. The height of the chair was set at 45 cm, which corresponds to the height of Si-St and St-Si of everyday people. Subjects performed two 30-second Si-St and St-Si tasks independently, with a 3-min rest interval, with their feet shoulder-width apart at a self-selected speed and hand posture (hands at the sides or clasped to the chest) at the beginning of the experiment. For each subject, five complete Si-St and St-Si tasks and durations were extracted from the two 30-second movement cycles collected for analysis.

The start and end points of Si-St and St-Si tasks were identified by the movement trajectories marked by the posterior superior iliac spine reflex on both sides. The Si-St motion begins when the reflective marker starts moving horizontally forward and ends when the reflective marker reaches its maximum vertical upward height (steady standing). The St-Si motion begins when the reflective marker starts moving downward from its maximum vertical height and ends when the reflective marker stops moving (steady sitting).

### Data processing

The MOtoNMS (a Matlab MOtion data elaboration Toolbox for NeuroMusculoSkeletal applications) [32] was used to generate OpenSim compatible files from the C3D files collected by the 3DMC system. The OpenSim (Version, opensim3.3, NCSRR, USA). The Inverse Kinematics tool of OpenSim was used to calculate sagittal joint angle of the hip, knee and ankle joint. The joint angles were filtered using a zero-lag fourth-order low-pass Butterworth filter with a cutoff frequency of 6 Hz based on recommendations of previous research [33–35]. The joint angle data were time-normalized to 100% of the motion cycle, in which 0% and 100% represent the start and the finish of Si-St or St-Si motion. The angle-angle diagrams of the hip-knee, knee-ankle and hip-ankle joint angles were ploted and the geometric characteristics including perimeter, area, and perimeter with area dimensionless ratio were derived using a customized Matlab code.

**Fig. 2:**
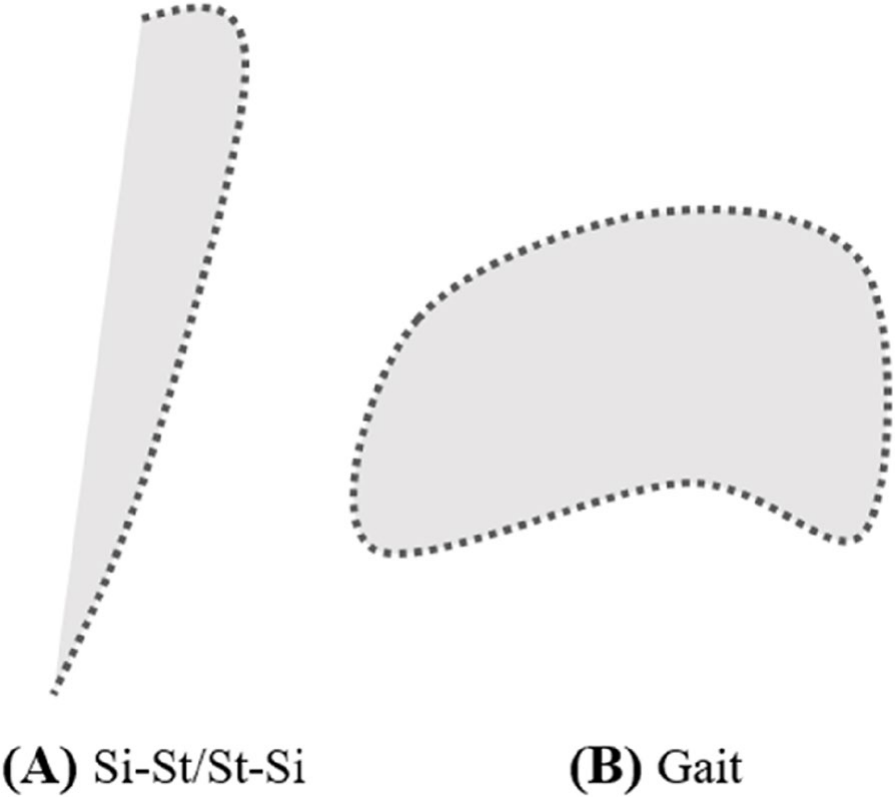
Angle-angle diagram (**A**) sit-to-stand and stand-to-sit movements schematic diagram; **B** gait movement schematic diagram. The shaded part represents the area

The perimeter $L$ is the total length of the trajectories of the angle-angle diagrams, calculated using Eqs. (1) and (2) [28, 30]. The area $A$ is defined as the region enclosed by the angle-angle curve and the straight line connecting the start and end points of that curve using Eq. (3), i.e., the area of the shaded part in Fig. 2A. To better study both the Si-St and St-Si movements in stroke patients with spastic hemiplegia, we divided the entire “Si-St-Si” movement into distinct Si-St and St-Si phase. As a result, the angle-angle diagram for each phase does not resemble the circular angle-angle diagram typically observed for a complete gait cycle [30, 36], as depicted in Fig. 2B.

Those parameters (perimeter and area) represent the conjoint range of angular motion of the hip-knee, hip-ankle and knee-ankle joints. The dimensionless ratio (DR) is defined as the perimeter divided by the square root of the area, calculated by Eq. (4) (26, 29). The variability in Si-St and St-Si movements was assessed by the coefficient of variance (CV) of the hip-knee, knee-ankle and hip-ankle inter-joints coordination parameters (i.e., perimeter and area), calculated by Eq. (5).

1 $$Li=\sqrt{{({\theta }_{ai}-{\theta }_{ai+1} )}^{2}+{({\theta }_{bi}-{\theta }_{bi+1} )}^{2}}$$

2 $$L={\sum }_{i}{L}_{i}$$

3 $$A=\frac{1}{2}{\sum }_{i}{(\theta }_{ai}{\theta }_{bi+1}-{\theta }_{ai+1}{\theta }_{bi})$$

4 $$DR=L\div\sqrt A$$

5 $$CV=\frac{{P}_{m}}{{P}_{\text{S}\text{D}}}$$

Where ${\theta }_{ai}$ and ${\theta }_{bi}$ represent the angles of two joints of interest at the time-step $i$, $Li$ is the length between time $i$ and $i$- 1, ${P}_{m}$ is the mean of the coordination parameters (i.e., perimeter or area) and ${P}_{\text{S}\text{D}}$ is the standard deviation of the coordination parameters.

The perimeter may increase as the area increases [26]. However, in some cases, even without a corresponding change in area, the lack of coordination can lead to an increase in perimeter [37]. A larger area usually indicates a greater angular range of motion experienced by a particular joint during a movement cycle [27, 37]. Additionally, the lower of the dimensionless ratio, the more regular the shape (e.g., not particularly elongated towards a specific direction) of the angle-angle diagram between the inter-joints of interest [26, 29].

### Statistical analysis

The Shapiro-Wilk test was used to examine the normal distribution of the data. For normally distributed data, the paired samples t-test was used to compare bilateral coordination differences, and the independent samples t-test was used to compare group (stroke versus healthy) differences in coordination parameters. For non-normally distributed data, the Wilcoxon signed rank test was used to compare bilateral coordination differences, and the Kruskal-Wallis test was used to compare group (stroke versus healthy) differences in coordination parameters and in age, height, body mass, body mass index (BMI), and foot length. Two-way ANOVA was used to examine the effect of movement duration and group (short versus longer duration) on coordination. All analyses were performed with SPSS 26.0 software (IBM/SPSS Inc., Armonk, NY, USA). The significance level was set at α = 0.05.

## Results

### Participants

A total of 26 subjects were studied. The demographic and clinical characteristics of all participants are shown in Table 1. The demographic characteristics, including age, sex, height, weight, BMI, and foot length were similar (*P*>0.05) between the stroke group and the healthy group. There were significant differences (*P*<0.001) between the groups in the duration of the Si-St and St-Si movements. Additionally, all subjects in the stroke group were accompanied by symptoms of spasticity (MAS scores>1).

**Table 1:** Anthropometric and clinical features of participants. Values are expressed as mean (SD)

	Stroke group (*N* = 13)	Healthy group (*N* = 13)	*P* valve
Age (years)	60.46 (5.39)	60.85 (5.93)	0.857
Sex (M/F)	11/2	8/5	0.185
Height (cm)	168.15 (6.88)	165.12 (4.36)	0.057
Weight (kg)	67.08 (10.92)	66.87 (6.31)	0.980
BMI (kg/m^2^)	23.62 (2.90)	24.51 (1.90)	0.158
Foot length (cm)	23.74 (1.80)	23.81 (1.08)	0.898
Total Si-St duration (s)	1.70 (0.54)	0.77 (0.13)	*P* < 0.001
Total St-Si duration (s)	1.83 (0.51)	0.80 (0.12)	*P* < 0.001
Affected side (L/R)	5/8	-	-
MAS (scores, 1/1+/2)	7/4/2	-	-
FACS (scores)	4.05(0.97)	-	-
Brunnstorm (scores)	4.35(1.06)	-	-

*N* number of participants, *M* male, *F* female, *BMI* body mass index, *Si-St* sit-to-stand, *St-Si* stand-to-sit, *L* left side, *R* right side, *MAS* Modified Ashworth Scale, *FACS* Functional Ambulation Category Scale

### Inter-joint coordination parameters

**Fig. 3:**
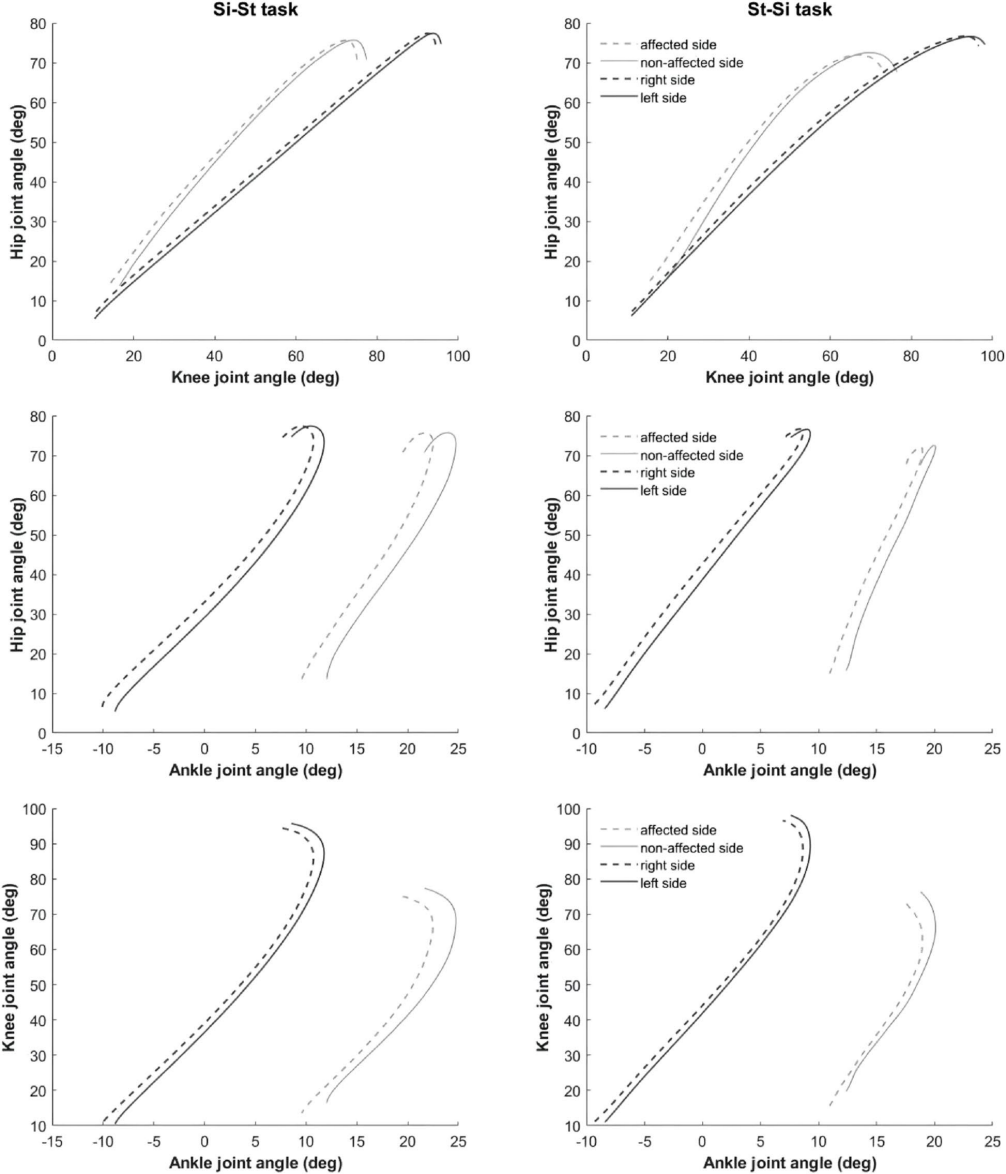
Mean angle-angle diagrams of bilateral hip-knee, hip-ankle, and knee-ankle inter-joints during the Si-St and St-Si tasks between the stroke group and the healthy group. The gray dashed and solid lines represent the affected and non-affected sides of the stroke group, and the black dashed and solid lines represent the right and left sides of the healthy group

The average angle-angle diagrams of the bilateral hip-knee, hip-ankle and knee-ankle inter-joints were calculated in the stroke group and the healthy group during Si-St and St-Si movements, as shown in Fig. 3. The patterns for the bilateral hip-knee, hip-ankle and knee-ankle inter-joint angle during the Si-St and St-Si movements in the stroke group are similar to that in the healthy group, which means that bilateral hip-knee, hip-ankle and knee-ankle inter-joint movements were coordinated in the stroke group.

The results of the stroke group regarding the parameters of inter-joint coordination during Si-St and St-Si movements and the comparison with the healthy group are summarized in Tables 2 and 3, respectively. There were no significant differences in the perimeter, area and dimensionless ratio of the bilateral hip-knee, hip-ankle and knee-ankle inter-joints during Si-St and St-Si tasks in the stroke group. The perimeter and area of the hip-ankle and knee-ankle inter-joints on the affected side are smaller than the non-affected side in the stroke group during Si-St and St-Si tasks, but the dimensionless ratio is greater than the non-affected side, showing that the shape of the angle-angle diagrams on the hemiplegic side tended to be less regular.

The perimeter values of the bilateral hip-knee and knee-ankle inter-joints during the Si-St and St-Si tasks in the stroke group were significantly different (*P* < 0.05) compared to the healthy group bilateral. The area of bilateral hip-ankle inter-joint diagrams and the dimensionless ratio of bilateral hip-knee inter-joint diagrams during the Si-St task, and the area of bilateral hip-knee inter-joint during the St-Si task in the stroke group are significantly lower (*P* < 0.05) than those in the healthy group. We also observed that the perimeter of all bilateral joint pairs and the area of the bilateral hip-ankle and knee-ankle angle-angle diagrams are smaller in the stroke group than in the healthy group. Interestingly, the area of the bilateral hip-knee diagram is larger in the stroke group than in the healthy group, indicating the greater conjoint range of angular motion of the hip-knee inter-joint.

**Table 2:** Comparison of inter-joint coordination parameters between the stroke and healthy groups during Si-St task. Values are expressed as mean (SD)

Task	Joints	Parameters	Stroke group		Healthy group	
			A	NA	R	L
	HK	Perimeter (deg)	105.86 (16.54) ^a^	105.99 (14.18) ^a^	121.51 (6.52)	122.26 (7.91)
		Area (deg^2^)	498.98 (215.77)	462.97 (194.46)	395.79 (305.32)	369.49 (294.91)
		DR	5.30 (2.25) ^a^	5.23 (1.22) ^a^	7.41 (2.66)	7.69 (2.61)
Si-St	HA	Perimeter (deg)	76.79 (15.88)	77.09 (13.75)	85.83 (4.64)	85.98 (5.85)
		Area (deg^2^)	175.35 (136.78)^a^	187.60 (87.16)^a^	291.27 (136.72)	290.03 (143.89)
		DR	7.14 (2.76)	5.95 (1.36)	5.46 (1.47)	5.69 (1.92)
	KA	Perimeter (deg)	77.31 (11.23)^a^	78.26 (8.94)^a^	95.75 (8.14)	95.95 (8.03)
		Area (deg^2^)	233.50 (130.49)	261.90 (114.94)	356.37 (178.89)	358.05 (171.16)
		DR	5.66 (1.59)	5.38 (1.99)	6.05 (2.85)	5.86 (2.30)

*Si-St* sit-to-stand, *HK* hip-knee, *HA* hip-ankle, *KA* knee-ankle, *deg* degree, *DR* dimensionless ratio, *A* affected sides, *NA* non-affected side, *R* right side, *L* left side

^a^The symbol indicates significant differences with the bilateral sides of the healthy subjects

**Table 3:** Comparison of inter-joint coordination parameters between the stroke and healthy groups during St-Si task. Values are expressed as mean (SD)

Task	Joints	Parameters	Stroke group		Healthy group	
			A	NA	R	L
	HK	Perimeter (deg)	104.92 (16.39)^a^	105.10 (14.80)^a^	115.61 (6.25)	117.31 (8.90)
		Area (deg^2^)	542.00 (191.86)^a^	504.65 (189.29)^a^	712.20 (268.16)	690.97 (256.39)
		DR	4.78 (1.42)	4.86 (0.85)	4.51 (0.68)	4.64 (0.71)
St-Si	HA	Perimeter (deg)	75.83 (15.47)	76.25 (13.81)	76.57 (6.22)	77.92 (7.35)
		Area (deg^2^)	151.87 (116.09)	169.88 (76.83)	212.48 (131.39)	146.40 (83.27)
		DR	7.50 (2.74)	6.34 (2.00)	5.88 (1.47)	7.25 (2.14)
	KA	Perimeter (deg)	75.91 (11.31)^a^	77.01 (9.63)^a^	88.71 (4.77)	90.84 (7.44)
		Area (deg^2^)	219.56 (113.44)	249.20 (110.48)	300.99 (160.94)	261.42 (104.86)
		DR	5.75 (1.96)	5.39 (1.78)	5.69 (1.69)	5.90 (1.07)

*St-Si* stand-to-sit, *HK* hip-knee, *HA* hip-ankle, *KA* knee-ankle, *deg* degree, *DR* dimensionless ratio, *A* affected sides, *NA* non-affected side, *R* right side, *L* left side

^a^The symbol indicates significant differences with the bilateral sides of the healthy subjects

### Effect of movement duration on inter-joint coordination parameters

The effect of movement duration on inter-joint coordination in stroke patients during the Si-St and St-Si tasks is demonstrated in Table 4. There was no significant difference (*P*>0.05) in bilateral hip-knee, hip-ankle and knee-ankle inter-joint coordination parameters between the two sub-groups of stroke patients with different movement durations during the Si-St and St-Si tasks. Although no statistical difference was found, we observed that except for the hip-ankle and knee-ankle areas, which increased with increasing movement duration during the St-Si task, the of the hip-knee, hip-ankle, and knee-ankle coordination parameters decreased with increasing movement duration in the Si-St and St-Si tasks in stroke patients.

**Table 4:** Effect of Si-St and St-Si duration on inter-joint coordination parameters in stroke patients. Values are expressed as mean (SD)

Tasks	Joints	Parameters	Short-duration (*n* = 6)		Long-duration (*n* = 7)	
			A	NA	A	NA
Si-St	HK	Perimeter (deg)	111.44 (17.67)	110.55 (15.58)	101.08 (15.14)	102.08 (12.70)
		Area (deg^2^)	499.29 (188.63)	477.89 (196.68)	498.72 (251.92)	450.19 (207.32)
	HA	Perimeter (deg)	82.61 (15.93)	81.23 (14.07)	71.81 (15.16)	73.55 (13.47)
		Area (deg^2^)	200.42 (140.03)	203.31 (69.27)	153.86 (141.10)	174.13 (103.62)
	KA	Perimeter (deg)	79.56 (13.73)	79.64 (11.52)	75.39 (9.27)	77.07 (6.76)
		Area (deg^2^)	248.68 (140.71)	258.99 (132.73)	220.49 (130.88)	264.39 (108.28)
St-Si	HK	Perimeter (deg)	110.85 (17.60)	111.28 (15.00)	99.85 (14.63)	99.80 (13.41)
		Area (deg^2^)	619.44 (193.92)	611.34 (190.80)	475.63 (176.49)	413.21 (142.07)
	HA	Perimeter (deg)	82.04 (15.18)	82.04 (12.98)	70.51 (14.67)	71.29 (13.38)
		Area (deg^2^)	143.53 (129.76)	156.71 (78.38)	159.01 (113.10)	181.17 (79.77)
	KA	Perimeter (deg)	78.24 (14.42)	78.79 (12.20)	73.92 (8.52)	75.48 (7.45)
		Area (deg^2^)	216.24 (128.43)	232.50 (133.16)	222.41 (109.42)	263.51 (95.50)

*Si-St* sit-to-stand, *St-Si* stand-to-sit, *HK* hip-knee, *HA* hip-ankle, *KA* knee-ankle, *deg* degree, *A* affected sides, *NA* non-affected side

### The coefficient of variance (CV) for inter-joint coordination parameters

Table 5 demonstrates the variability of hip-knee, hip-ankle and knee-ankle diagrams parameters (including perimeter and area) during the of Si-St and St-Si tasks using the coefficient of variance (CV). The perimeter variabilities of the hip-knee, hip-ankle and knee-ankle diagrams are smaller on the affected side than on the non-affected side during the Si-St and St-Si tasks in the stroke group. There are significant differences (*P* < 0.05) in the CV of the hip-knee diagram perimeter during the Si-St task between the affected and non-affected side in the stroke group. The CV of the area of the bilateral hip-knee diagrams during the Si-St task is significantly decreased (*P* < 0.05) in the stroke group compared with the healthy group. The CV of the perimeter and area of the hip-ankle and knee-ankle diagrams during the Si-St and St-Si task were not significantly different between the affected side and the non-affected side of the stroke subjects or compared with the healthy group.

**Table 5:** The perimeter and area coefficient of variances (CV) of hip-knee, hip-ankle and knee-ankle diagrams between the stroke and healthy groups. Value are expressed as mean (SD)

Tasks	Joint	Parameters	Stroke group		Healthy group	
			A	NA	R	L
Si-St	HK	Perimeter (deg)	0.04 (0.01)^a^	0.05 (0.02)	0.04 (0.02)	0.04 (0.02)
		Area (deg^2^)	0.33 (0.27)^b^	0.35 (0.19)^b^	0.60 (0.34)	0.58 (0.31)
	HA	Perimeter (deg)	0.05 (0.02)	0.06 (0.03)	0.05 (0.02)	0.06 (0.02)
		Area (deg^2^)	0.50 (0.33)	0.39 (0.23)	0.44 (0.25)	0.50 (0.23)
	KA	Perimeter (deg)	0.04 (0.02)	0.06 (0.03)	0.05 (0.04)	0.05 (0.03)
		Area (deg^2^)	0.29 (0.20)	0.26 (0.16)	0.30 (0.20)	0.33 (0.16)
St-Si	HK	Perimeter (deg)	0.04 (0.03)	0.05 (0.03)	0.04 (0.02)	0.04 (0.02)
		Area (deg^2^)	0.46 (0.40)	0.42 (0.28)	0.37 (0.15)	0.37 (0.14)
	HA	Perimeter (deg)	0.07 (0.04)	0.07 (0.04)	0.07 (0.03)	0.06 (0.03)
		Area (deg^2^)	0.58 (0.16)	0.49 (0.25)	0.49 (0.26)	0.68 (0.25)
	KA	Perimeter (deg)	0.05 (0.03)	0.06 (0.03)	0.04 (0.03)	0.04 (0.02)
		Area (deg^2^)	0.35 (0.18)	0.32 (0.19)	0.41 (0.21)	0.38 (0.20)

*Si-St* sit-to-stand, *St-Si* stand-to-sit, *HK* hip-knee, *HA* hip-ankle, *KA* knee-ankle, *deg* degree, *A* affected sides, *NA* non-affected side, *R* right side, *L* left side

^a^The symbol indicates significant differences between the affected and non-affected sides in the stroke group

^b^The symbol indicates significant differences with the bilateral sides of the healthy subjects

## Discussion

The objective of this study was to investigate the coordination characteristics of the bilateral hip-knee, hip-ankle and knee-ankle diagrams in the sagittal plane in stroke patients with hemiplegia during Si-St and St-Si tasks and compare them with age-matched healthy adults, as well as examine the impact of duration on joint coordination. Our study highlights the importance of understanding joint coordination characteristics in stroke patients during Si-St and St-Si tasks and with different movement durations, which can help develop targeted rehabilitation programs to improve their motor functions.

Most studies have shown that Si-St and St-Si movements involve the transition between sitting and standing positions, predominantly involving flexion and extension activities of the lower limb joints within sagittal movements [38–40]. Specifically, in the sagittal plane, the ankle angle ranges from 0.8° to 26°, the knee angle ranges from − 7.5° to 100.6°, and the hip angle ranges from 5.6° to 89° [41]. In the coronal plane, the ankle, knee, and hip angles range from 0° to 7°, -2° to 44.7°, and − 9° to 0.3°, respectively. In the transverse plane, the ankle, knee, and hip joint angles range from − 33° to 1°, -34° to 27.5°, and 20° to 42°, respectively. The difference between the maximum and minimum values of the sagittal plane is about 100° at the knee and hip, and about 25° at the ankle. These results may suggest that the biggest problems in performing Si-St and St-Si movements in situations where joint mobility is limited will be evident in the sagittal plane [41]. Additionally, the widely accepted definition of Si-St and St-Si movements is also based on data derived from sagittal plane angles and force plates [42]. This definition involves the calculation of sagittal plane kinematic data (e.g., joint angles), ground reaction force data at specific time points, and the relative time interval between them. Therefore, focusing on sagittal plane information can provide valuable insights into Si-St and St-Si movements, particularly regarding joint coordination assessed through the sagittal plane angle-angle diagram.

Our results showed that there were no significant differences in coordination between the affected and non-affected side of the stroke patients at the sagittal plane hip-knee, hip-ankle and knee-ankle inter-joints, but there were significant inter-joint coordination differences between the stroke group and the healthy group. The bilateral motion patterns of the hip-knee, hip-ankle and knee-ankle inter-joints in the Si-St and St-Si tasks are similar between the stroke group and the healthy group, as demonstrated by angle-angle diagrams. However, the stroke group exhibited less conjoint angular motion of the hip-knee, hip-ankle, and knee-ankle joints, indicating limited coordination compared to the healthy individuals. Furthermore, while the perimeter and area of the hip-knee, hip-ankle and knee-ankle joints did not differ significantly between the short-duration and long-duration sub-groups in stroke patients, some coordination parameters decreased as duration increased. In the stroke patients, we found significant bilateral differences only in the hip-knee diagram perimeter coefficient of variation (CV) during the Si-St task. The bilateral hip-knee diagram area CV during the Si-St task was the only parameter with significant difference between the stroke group and healthy group.

A detailed analysis of the hip-knee, hip-ankle and knee-ankle coordination suggests that stroke patients exhibit similar shape of the mean angle-angle diagrams to those of healthy subjects, but with a relatively small conjoint range of angular motion, especially on the affected side, during Si-St and St-Si tasks. Impaired subjects typically develop compensatory movement strategies by simultaneously implementing a set of basic compensations [43]. In this case, stroke patients with hemiplegia rely more on the non-affected side to complete the activity during the Si-St and St-Si tasks [44]. This is further supported by the finding that the perimeter and area of the hip-ankle and knee-ankle diagrams of the non-affected side were larger than those of the affected side, indicating that the non-affected side had more conjoint movement during the Si-St and St-Si tasks. As confirmed by the angle-angle diagrams, we also found that stroke patients had limited ankle angle during Si-St and St-Si tasks, and therefore the hip-ankle and knee-ankle coordination involving the ankle joint is limited and less smooth. Additionally, the dimensionless ratio parameters of the hip-ankle and knee-ankle diagrams are greater than that of hip-knee diagram, indicating a less smooth shape.

Walking can be considered as a motor task that requires bilateral coordination between two unilateral rhythmic movements [45]. Similarly, Si-St and St-Si movements also require bilateral coordination movements. Our results showed that stroke patients with hemiplegia had poorer bilateral inter-joint coordination than healthy individuals, particularly in the ankle joint during the Si-St task. This suggests that joint angles during functional activities is a significant factor influencing coordination parameters, which are calculated by analyzing the trajectories of the angle of one joint with other joints [27]. Furthermore, stroke patients had limited the bilateral knee-ankle and hip-ankle inter-joint coordination during the St-Si task compared to the Si-St task. This implies a higher risk of falls due to uncoordinated joint movements, particularly for stroke patients with longer Si-St and St-Si durations. Longer durations of these tasks have been linked to decreased muscle strength [46–48] and poorer balance in stroke patients [49], leading to impaired joint coordination and increased risks of falling [50]. Our findings suggest that stroke patients may benefit from interventions targeting joint coordination during Si-St and St-Si tasks, particularly for those with longer task durations.

In addition, it’s worth noting that falls are not solely influenced by the lower limbs; other factors such as trunk characteristics [51]. The trunk is crucial for maintaining body stability and balance during Si-St and St-Si movements. When the body moves in any plane, the trunk makes corresponding movements to compensate for the change in body’s center of mass (COM) [52]. In the Si-St movement performed by healthy individuals, the initiation of trunk flexion precedes knee extension, whereas in the St-Si movement, trunk flexion and the knee flexion occur almost simultaneously [53]. This requires the coordinated control of the body’s COM for both anterior-posterior and vertical displacement [53]. However, stroke patients with hemiplegia experienced decreased functional performance during Si-St and St-Si movements due to weakness of the trunk flexor and extensor muscles, as well as decreased strength on the affected side [54]. Additionally, individuals who have experienced falls exhibit greater range of motion (ROM) in the trunk segment during Si-St flexion and extension phases, less ROM in hip joint extension, and greater medial-lateral center of mass (ML-COM) trajectories [24]. In summary, the coordination between the trunk and lower limbs is crucial for maintaining balance and stability during these movements.

Coordination is recognized as an important factor affecting postural stability [55], particularly in the context of inter-joint coordination variability following fatigue [21, 56, 57]. While previous studies have reported on the variability of inter-joint coordination during gait in stroke patients [58], little research has explored variability during the Si-St and St-Si tasks. Our result indicated that the CV of hip-knee, hip-ankle, and knee-ankle joints in stroke patients during the St-Si task was greater than during the Si-St task, potentially due to differences in motor control types, muscle coordination, and sensory input [59]. Interestingly, Only the CV of the hip-knee diagram area during the Si-St task was significantly smaller in the stroke group than in the healthy group. This finding may be attributed to limited knee joint angle in stroke patients, as supported by the angle-angle diagrams.

The results of this study offer novel insights into the inter-joint coordination of bilateral hip-knee, hip-ankle and knee-ankle joints during the Si-St and St-Si tasks in stroke patients. The coordination parameters, which include perimeter, area, and dimensionless ratio, showed that the bilateral hip-knee, hip-ankle and knee-ankle joints were coordinated in stroke patients. However, inter-joint coordination on the affected side was limited, and there were differences in inter-joint coordination between the stroke group and the healthy group during the Si-St and St-Si tasks. Furthermore, our findings suggest that the duration of Si-St and St-Si tasks has an impact on joint coordination in stroke patients, as coordination decreased with increasing task duration. However, this may need to be confirmed by including more sample size. It is worth noting that these results are based on a relatively small sample size, and further investigation with a larger sample is needed to confirm our findings. It is also important to acknowledge that the coordination parameters used in this study were derived from the geometric characteristics of the joint angles [27], whereas other phase analysis method [21, 60, 61], such as those based on the phase angle difference between distal and proximal joint, may yield different results. In addition, it should be noted that our results may not be generalizable to patients with severe stroke, because our included stroke patients had a higher level of function, as indicated by FACS and Brunnstorm Scale scores of more than four levels.

This study has several limitations that should be acknowledged. Firstly, we set the same chair height (45 cm) for all subjects, which may influence the lower limb kinematics of subjects with different body height. Secondly, although significant differences between groups were reported, the sample size was small, which may limit the generalizability of the findings. Lastly, we only investigated the lower extremity joint coordination based on sagittal plane kinematics. We will generalize our study other planes (i.e., the coronal and transverse plane) in patients with stroke hemiplegia during Si-St and St-Si movements in our future research.

## Conclusion

This study investigated the inter-joint coordination of bilateral hip-knee, hip-ankle, and knee-ankle joints during the Si-St and St-Si tasks in stroke patients. Our findings indicate that stroke patients exhibit limited inter-joint coordination on the hemiplegic side during these tasks compared to healthy individuals. We also found that coordination tended to decrease with increasing duration, which may have implications for rehabilitation interventions. Overall, our study contributes to a better understanding of the inter-joint coordination patterns during Si-St and St-Si tasks in stroke patients, which can potentially inform the development of more targeted and effective rehabilitation programs.

## Data Availability

The dataset supporting the conclusions of this article is available in the [Science Data Bank] repository, [hyperlink to dataset in https://www.scidb.cn/s/nEnia2].

## Abbreviations

Si-St: Sit-to-stand
St-Si: Stand-to-sit
CV: Coefficient of variation
FACS: Functional Ambulation Category Scale
MAS: Modified Ashworth Scale
